# The Preventive Effects of Probiotic *Prevotella histicola* on the Bone Loss of Mice with Ovariectomy-Mediated Osteoporosis

**DOI:** 10.3390/microorganisms11040950

**Published:** 2023-04-06

**Authors:** Yuan-Wei Zhang, Mu-Min Cao, Ying-Juan Li, Ren-Wang Sheng, Ruo-Lan Zhang, Meng-Ting Wu, Jia-Yu Chi, Rui-Xin Zhou, Yun-Feng Rui

**Affiliations:** 1 Department of Orthopaedics, Zhongda Hospital, School of Medicine, Southeast University, Nanjing 210009, China; 2 Multidisciplinary Team (MDT) for Geriatric Hip Fracture Management, Zhongda Hospital, School of Medicine, Southeast University, Nanjing 210009, China; 3 School of Medicine, Southeast University, Nanjing 210009, China; 4 Orthopaedic Trauma Institute (OTI), Southeast University, Nanjing 210009, China; 5 Trauma Center, Zhongda Hospital, School of Medicine, Southeast University, Nanjing 210009, China; 6 Department of Geriatrics, Zhongda Hospital, School of Medicine, Southeast University, Nanjing 210009, China

**Keywords:** probiotics, *Prevotella histicola*, osteoporosis, bone loss, ovariectomy

## Abstract

It has been demonstrated that the disturbance of gut microbiota (GM) is closely related to the reduction of bone mass and incidence of osteoporosis (OP). The aim of this study is to investigate whether the supplementation of *Prevotella histicola* (Ph) can prevent the bone loss in mice with ovariectomy (OVX)-mediated OP, and further explore relevant mechanisms. Regular (once a day for 8 consecutive weeks) and quantitative (200 µL/d) perfusion of Ph (the bacteria that orally gavaged) was conducted starting from 1 week after the construction of mice models. Bone mass and bone microstructure were detected by Micro-computed tomography (Micro-CT). Expressions of intestinal permeability, pro-inflammatory cytokines, and osteogenic and osteoclastic activities of mice were analyzed by histological staining and immunohistochemistry (IHC). 16S rRNA high throughput sequencing technique was applied to analyze the alterations of composition, abundance, and diversity of collected feces. Regular and quantitative perfusion of Ph mitigated the bone loss in mice with OVX-mediated OP. Compared with OVX + PBS group, perfusion of Ph repressed osteoclastogenesis and promoted osteogenesis, reduced release of pro-inflammatory cytokine cytokines (interleukin-1β (IL-1β) and tumor necrosis factor-α (TNF-α)), and reversed expressions of tight junction proteins (zonula occludens protein 1 (ZO-1) and Occludin). Besides, the perfusion of Ph improved the composition, abundance, and diversity of GM. Collectively, this study revealed that regular and quantitative perfusion of Ph can improve the bone loss in mice with OVX-mediated OP by repairing intestinal mucosal barrier damage, optimizing intestinal permeability, inhibiting release of pro-osteoclastogenic cytokines, and improving disturbance of GM.

## 1. Introduction

With the alteration of global population structure and the acceleration of population aging, osteoporosis (OP) and OP-related fragile fractures have become a public health issue that concerned the whole society [1,2]. OP is a systemic bone disease characterized by the low bone mass and destruction of bone microstructure, which results in increased bone fragility, decreased bone strength, and enhanced risk of fractures [3,4,5]. It has been reported in previous studies that about 20% of female and 7% of male over the age of 50 might be affected by OP, and half of them may experience the osteoporotic fractures in their later life [6,7,8]. Although various drugs with different mechanisms have been applied to clinic, most patients with OP have not received accurate, scientific, and comprehensive treatment due to the cost-effectiveness and potential side effects of currently available drugs [9,10]. Hence, in view of current dilemma, more and more attention should be focused on the scientific intervention and the exploration of novel approaches of OP in the future clinical practice and basic researches [11].

Gut microbiota (GM) is a collection of microorganisms “that” colonized the human gut, which contains about 10 trillion bacteria, and the total number of genes in microbiota is 150 times that the human genome [12]. As the largest microbial repository in human body, the dynamic balance of intestinal microecology is closely related to the human health [13]. Moreover, the abundance and diversity of GM may gradually decrease with advancement of age [14], with a dysbiotic pattern increasing harmful bacteria, while decreasing the number of beneficial bacteria. Several studies in recent years have also indicated that there are certain differences between the GM of patients with OP and healthy individuals, and the severity of bone loss in human body is also closely related to the alterations of GM [15,16], which to some extent reveals that GM might be a potential target for the prevention and treatment of OP.

Probiotics are defined as the active microorganisms beneficial to health, which can stimulate the activity of carbohydrate bacteria, enhance the secretion of organic acids, improve the intestinal acidic microenvironment, increase intestinal antibacterial ability, and inhibit the growth and reproduction of pathogenic bacteria, thereby maintaining the intestinal function and GM balance [17]. Meanwhile, previous studies have also shown that probiotic products can influence bone metabolism by modulating GM, and the anti-OP potential of some partial probiotic strains have also been verified in a variety of OP-related animal models [18,19,20]. *Prevotella histicola* (Ph) is one kind of probiotic closely associated with human diet habits, and its potential anti-OP effects have gradually raised scientific interest. Ph is rich and diverse in the human microbiome, distributed in different organs and ecological niches, such as the oral cavity, gut, and vagina. Diet, lifestyle, age, gender, and geographical location may affect the diversity, specificity, and function of Ph [21,22]. In a previous study, Wang et al. [23] collected the feces from 42 individuals (24 patients with postmenopausal OP and 18 women with normal bone mass) for 16S rRNA sequencing, and then observed that the α-diversity was decreased in the GM of patients with postmenopausal OP, the composition structure of GM was altered, and the abundance of Ph was also reduced, which showed the protective potentials of Ph for OP. Meanwhile, this study also verified that Ph may contribute to reducing the activity of osteoclasts and inhibiting the increase of osteoclastogenic factors in both serum and bone. More importantly, in a recent study conducted by our research group [24], we revealed that fecal microbiota transplantation (FMT) played an active role in remodeling the GM of mice with ovariectomy (OVX)-mediated OP and improving the bone loss, and Ph was also one of the key differentiators in this process (the GM with highest abundance expression). Based on this, the purpose of this current research was to investigate whether the supplementation of Ph could prevent the bone loss in the mice with OVX-mediated OP, and further explore its relevant mechanisms.

## 2. Materials and Methods

### 2.1. Ethics, Preparation and Disposal Related to Animal Experiments

The female C57BL/6 mice aged 8 weeks were acquired from Aniphe Biolaboratory (Nanjing, Jiangsu, China) and housed under specific pathogen-free (SPF) conditions (food and water were available ad libitum; breeding environment were maintained at 25 ± 2 °C and 50 ± 5% humidity; with a 12 h:12 h light/dark cycle). Following a one-week period of environment acclimatization, the mice were then used for experiments. The guidelines for care and use of laboratory animals of Southeast University were used to guide animal experiment design schemes, and the institutional animal care and use committee (IACUC) of School of Medicine, Southeast University approved the overall animal studies (approval number: 20210510028).

### 2.2. Construction of Mice Models with OVX-Mediated OP

The construction of mice models with OVX-mediated OP was conducted with the reference to the schemes in previous literatures [25,26]. Mice were anesthetized with 1% pentobarbital sodium solution (40 mg/kg) and placed on the sheet in a prone position. After removing the hair on the back of mice, the skin of the midline of back was selected for a longitudinal incision (approximately 1 cm). Then, the muscle fibers of back were separated layer by layer through the tip of tissue scissors, and then the bilateral ovaries of mice were located, identified, ligated, and resected successively. The cotton swabs were used to stop bleeding and the incisions were closed layer by layer with absorbable sutures, and the mice were then placed on a warm blanket and waited for spontaneous resuscitation.

### 2.3. Animals Grouping and Sample Extraction

**Figure 1:**
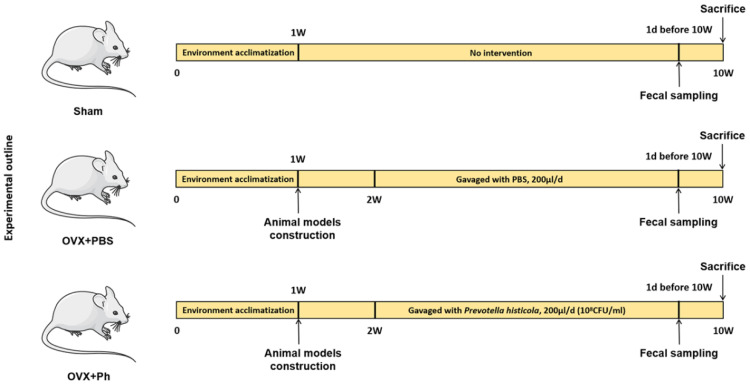
The whole experimental outline of this current study.

The animals grouping and experimental progress were shown in Figure 1. Specifically, mice were randomly divided into three groups, including the Sham group (*n* = 6), OVX + phosphate buffer solution (PBS) group (*n* = 6) and OVX + Ph group (*n* = 6). Therein, the mice in Sham group underwent the same procedures as described above, except that bilateral ovarian tissues were retained. More importantly, considering that the co-caged fecal feeding behaviors of mice may obscure the therapeutic effects of microbial-related interventions to certain extents [27,28], each mouse in each group was housed in a single cage and received the corresponding periodic interventions. Moreover, starting from one week after the construction of mice models with OVX-mediated OP, the mice in OVX + PBS group and OVX + Ph group were orally gavaged with PBS and Ph bacteria correspondingly (both 200 μL) once a day for 8 consecutive weeks [29]. Then, on the day before mice were sacrificed and after the completion of last perfusion, mice in each group were deprived of right to ingest food and water (12 h), and fresh feces of mice were collected and immediately stored at −80 °C. On the last day of experimental cycle, the mice in each group were sacrificed by inhaling excessive carbon dioxide, and the femur and colon tissues of mice in each group were extracted for further detection.

### 2.4. Culture and Preparation of Ph

The strain freeze-dried powder of Ph was acquired from BeNa Biological Company (Xinyang, Henan, China). During the process of culture, the lyophilized tube containing strain freeze-dried powder of Ph was taken out, and 0.5 mL sterile water that had been balanced in an anaerobic environment for 24 h was inhaled and then injected into the lyophilized tube. Subsequently, once the strain freeze-dried powder of Ph was fully dissolved, the solution was injected into the blood plates and evenly coated. The blood plates were then placed in an anaerobic environment at 37 °C, cultured for one to two generations (24 to 48 h), and the vitality of Ph strain could be restored [30]. After the activity of Ph strain was restored, the medium containing Ph strain was transferred to a centrifuge tube and then centrifuged at 4 °C and 4000× *g* rpm/min for 10 min. After the centrifugation, the supernatant was filtered through a 0.22 µm filter membrane for two to three times to obtain the supernatant of Ph, which was then used for perfusion (by gavage) of mice in OVX + Ph group. Of note, the centrifugation and filtration operations described above were mainly targeted at impurities during transfer process to maximize the removal of impurities and ensure the effectiveness of the gavage of Ph.

### 2.5. The Detection of Micro-Computed Tomography (Micro-CT)

On the last day of experimental cycle, the mice in each group were sacrificed and the femur tissues were extracted for the detection of Micro-CT. In details, the Micro-CT scanning equipment (Bruker AXS, Karlsruhe, Germany) with the especial operating parameters (voltage: 70 kV, current: 200 µA, resolution: 9 µm) was applied for analysis. After the completion of scanning, the data was acquired and saved. We then used the NRecon software (Bruker AXS, Karlsruhe, Germany) to reconstruct scanned images, and built the visual three-dimensional (3D) femoral models via CTVol software (Bruker AXS, Karlsruhe, Germany). Furthermore, a morphometric analysis was conducted on the 50 cross-sections (0.5 mm) of region of interest (ROI) under femoral growth plate, and a further analysis was performed on 200 cross-sections of the trabecular bone with a height of 2 mm. The bone tissue parameters of ROI were then assessed by the CTAn program (Bruker AXS, Karlsruhe, Germany), which included the bone mineral density (BMD), bone surface area/total volume (BS/TV), bone volume/total volume (BV/TV), bone surface/volume ratio (BS/BV), trabecular number (Tb.N), trabecular distance (Tb.Sp), trabecular thickness (Tb.Th) and structure model index (SMI).

### 2.6. Histological Staining and Assessment

The femur and colon tissues extracted from the mice in each group were immersed in 4% paraformaldehyde and fixed for 24 h, then the 12.5% ethylenediaminetetraacetic acid (EDTA) was used to decalcify the femur tissues for three to four weeks. Next, the decalcified femur tissues and fixed colon tissues were embedded in paraffin to prepare 5 µm-thick tissue sections. Subsequently, the prepared femur and colon tissue sections were stained with hematoxylin eosin (H&E), and the generation of osteoclasts in femur tissue sections was assessed by the tartrate resistant acid phosphatase (TRAP) staining. Based on this, the number of osteoclasts was then quantitatively assessed by the Image J software (National Institutes of Health, Bethesda, MD, USA).

### 2.7. Immunohistochemistry (IHC) and Assessment

After the femur and colon tissue sections were balanced in the 0.1 M tris-buffered normal saline for 10 min, the sections were then placed into the PBS and blocked with 10% normal goat serum for 1 h. Subsequently, the colon tissue sections were incubated overnight at 4 °C with the primary antibodies of Occludin, zonula occludens protein 1 (ZO-1), interleukin-1β (IL-1β), and tumor necrosis factor-α (TNF-α), and femur tissue sections were incubated with primary antibodies of osteopontin (OPN), osteoprotegerin (OPG), receptor activator for nuclear factor-κB ligand (RANKL), IL-1β, TNF-α, and recombinant runt related transcription factor 2 (RUNX2). Next, the tissue sections were then rinsed in PBS for about 15 min, and further incubated with the secondary antibody bound with horseradish peroxidase at room temperature for about 1 h. The expression of different markers was then visualized microscopically with 3,3’-diaminobenzidine, and the quantitative analysis was conducted using Image J software (National Institutes of Health, Bethesda, MD, USA) in six randomly selected high-power fields (HPFs) revealing positive cells regions.

### 2.8. 16S rRNA High Throughput Sequencing and Bioinformatics Assessment

The fresh feces were collected on the day before mice were sacrificed and after the completion of last perfusion, and immediately stored at −80 °C. The HiPure Stool DNA Kits (Magen, Guangzhou, China) were applied to extract total DNA, after which the conserved 16S V3-V4 rRNA regions (V3: 341F, CCTACGGGNGGCWGCAG and V4: 806R, GGACTACHVGGGTATCTAAT) was amplified through the polymerase chain reaction (PCR). Next, the amplified products were further detected by electrophoresis on the agarose gel with a concentration of 2%, and sequenced by the Illumina platform (Illumina, San Diego, CA, USA). The microbiome analysis part of this study was undertaken and implemented by the LC-Bio (Hangzhou, Zhejiang, China). After the completion of sequencing, overlap was used to splice the paired-end data, the quality control was performed, and the chimeric filtering was conducted to obtain high-quality data. The obtained sequences were then clustered to obtain the operational taxonomic units (OTUs) for further diversity analysis, species classification annotation, and difference analysis. Based on the species annotations, the columnar accumulations of abundance of samples in each group were then selected at the taxonomic levels of phylum, class, order, family, genus, and species. In accordance with the results of species annotations, top ten OTUs with maximum abundance of each group at each taxonomic level were selected to build columnar accumulation graphs of relative abundance, thus observing the abundance of each group at different taxonomic levels. The subsequent α-diversity analysis, β-diversity analysis, significant species difference analysis, correlation analysis, compositions and difference analysis, function prediction analysis and so on, can excavate differences between groups. The α-diversity analysis mainly evaluated the diversity within the country via several indexes, such as Shannon, Simpson, and Chao1. The SILVA database and NT-16S database are used for species annotation, and the abundance of each species in each sample was calculated. Based on the obtained statistical information of species abundance, difference analysis was conducted among the comparison groups. The different statistical methods were selected according to the specific sample conditions. Fisher’s exact test was used to compare the differences of samples without biological duplication; Mann-Whitney U test was used to compare the difference between two groups of samples with biological replicates; Kruskal-Wallis test, comparing the groups with biological replicas, and the screening threshold: *p* < 0.05. PICRUSt tool was applied to assess the different pathways of samples in each group through the kyoto encyclopedia of genes and genomes (KEGG) metabolic pathway.

### 2.9. Statistical Evaluation

The data in this present study were presented as mean ± standard deviation (SD), and the statistical evaluation was performed by the SPSS 26.0 software (IBM, Chicago, IL, USA). The data between two groups were analyzed by the means of *t*-test, and the data between multiple groups were analyzed by the means of one-way ANOVA test. Therein, each experiment included at least three repeated results, and a *p* value less than 0.05 was regarded statistically significant.

## 3. Results

### 3.1. The Perfusion of Ph Mitigated the Bone Loss in Mice with OVX-Mediated OP

**Figure 2:**
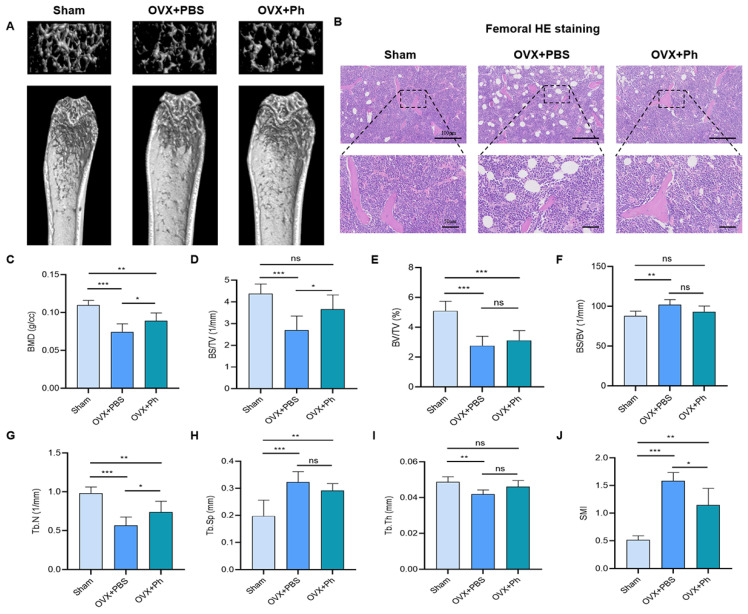
The perfusion of Ph mitigated the bone loss in mice with OVX-mediated OP. A morphometric analysis was conducted on the 50 cross-sections (0.5 mm) of ROI under femoral growth plate, and a further analysis was performed on 200 cross-sections of the trabecular bone with a height of 2 mm. (**A**) The Micro-CT scanning results of the distal femur of the mice in different group; (**B**) The femoral H&E staining results of the distal femur of the mice in different group. The scale bars represented as 100 μm and 50 μm; (**C**) BMD; (**D**) BS/TV; (**E**) BV/TV; (**F**) BS/BV; (**G**) Tb.N; (**H**) Tb.Sp; (**I**) Tb.Th; (**J**) SMI of femoral trabecular bone were analyzed by Micro-CT. * *p* < 0.05, ** *p* < 0.01, and *** *p* < 0.001 compared with comparable group; ns no significance.

**Figure 3:**
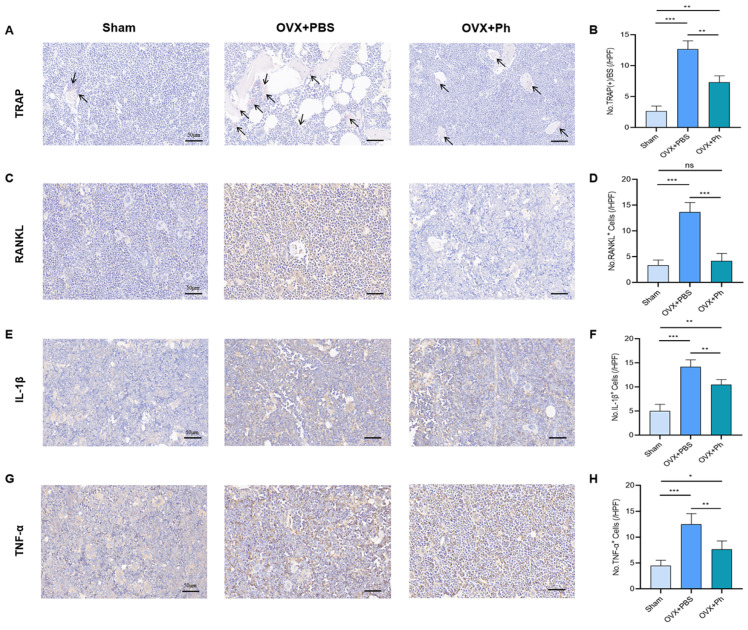
The perfusion of Ph repressed the osteoclastogenesis. (**A**) The TRAP stained the sections of distal femur tissues. The scale bars represented as 50 μm; (**B**) No. TRAP^+^ cells; (**C**) The RANKL stained the sections of the distal femur tissues. The scale bars represented as 50 μm; (**D**) No. RANKL^+^ cells; (**E**) The IL-1β stained the sections of distal femur tissues. The scale bars represented as 50 μm; (**F**) No. IL-1β^+^ cells; (**G**) The TNF-α stained the sections of distal femur tissues. The scale bars represented as 50 μm; (**H**) No. TNF-α^+^ cells. * *p* < 0.05, ** *p* < 0.01, and *** *p* < 0.001 compared with comparable group; ns no significance.

We applied Micro-CT scanning to detect the structure of distal femur of mice in each group after the completion of experimental cycle, and selected 50 cross-sections (0.5 mm) under distal femur growth plate as ROI regions for quantitative morphometric analysis. The results revealed that compared with morphology of distal femur of mice in Sham group, the cortical bone of mice in OVX + PBS group was thinner, the cancellous bone was sparser, and the number of bone trabeculae was decreased. However, after the intervention of regular (once a day for 8 consecutive weeks) and quantitative (200 µL) perfusion of Ph (the bacteria that orally gavaged), the loss of cancellous bone of mice in OVX + Ph group was ameliorated, and the number of bone trabeculae was reserved (Figure 2A). Similarly, the results of femoral H&E staining (Figure 2B) and TRAP staining (Figure 3A,B) also provided such research evidence. Furthermore, the results of the analysis of bone tissue parameters (Figure 2C–J) suggested that compared with mice in OVX + PBS group, the values of BMD (*p* < 0.05), BS/TV (*p* < 0.05), Tb. N (*p* < 0.05) and SMI (*p* < 0.05) in ROI region of mice in OVX + Ph group were enhanced, while the values of BV/TV, BS/BV, Tb. SP and Tb. Th were not significantly different (*p* > 0.05). In addition, compared with the mice in Sham group, the ROI region of mice in OVX + Ph group mice also had statistical differences regarding the values of BMD (*p* < 0.01), BV/TV (*p* < 0.001), Tb. N (*p* < 0.01), Tb. SP (*p* < 0.01) and SMI (*p* < 0.01).

**Figure 4:**
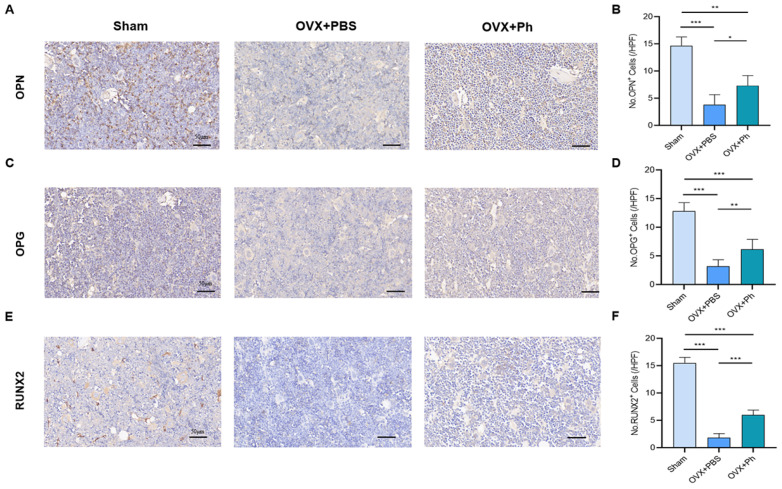
The perfusion of Ph facilitated the osteogenesis. (**A**) OPN stained the sections of distal femur tissues. The scale bars represented as 50 μm; (**B**) No. OPN^+^ cells; (**C**) The OPG stained the sections of distal femur tissues. The scale bars represented as 50 μm; (**D**) No. OPG^+^ cells; (**E**) The RUNX2 stained the sections of distal femur tissues. The scale bars represented as 50 μm; (**F**) No. RUNX2^+^ cells. * *p* < 0.05, ** *p* < 0.01, and *** *p* < 0.001 compared with comparable group.

In terms of IHC and assessment, the results revealed that the expression of RANKL increased after the OVX, and the regular and quantitative perfusion of Ph reduced the expression of RANKL relative to that in the OVX + PBS group (Figure 3C,D). Related to this was that the expression of IL-1β and TNF-α increased after OVX, whereas the regular and quantitative perfusion of Ph also reduced the expressions of IL-1β and TNF-α in femur tissue (Figure 3E–H). Similarly, the relevant results also indicated that the expressions of OPN, OPG and RUNX2 decreased after OVX, and regular and quantitative perfusion of Ph enhanced the expressions of OPN, OPG and RUNX2 relative to that in OVX + PBS group (Figure 4). Combined with above findings, we explored the role of the perfusion of Ph in relieving OVX-mediated OP, and it may be related to the reduction of pro-inflammatory cytokines level to achieve the balance between bone resorption and bone formation.

### 3.2. The Perfusion of Ph Reversed OVX-Mediated Intestinal Mucosal Barrier Damage

**Figure 5:**
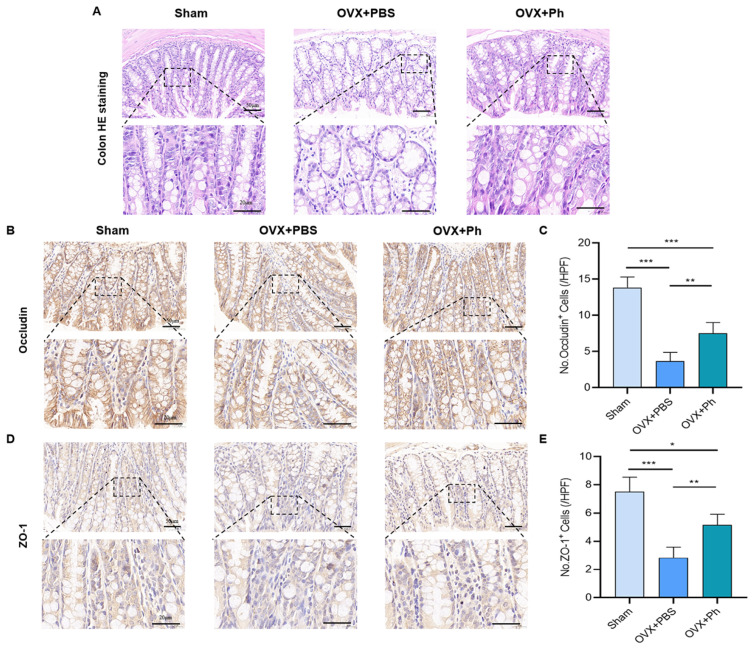
The perfusion of Ph reversed the OVX-mediated intestinal mucosal barrier damage. (**A**) The H&E staining of colon tissues. The scale bars represented as 50 μm and 20 μm; (**B**) Occludin stained the sections of colon tissues. Scale bars represented as 50 μm and 20 μm; (**C**) No. Occludin^+^ cells; (**D**) The ZO-1 stained the sections of colon tissues. The scale bars represented as 50 μm and 20 μm; (**E**) No. ZO-1^+^ cells. * *p* < 0.05, ** *p* < 0.01, and *** *p* < 0.001 compared with comparable group.

In Figure 5A, it could be noticed that OVX-mediated estrogen-deficiency was closely associated with intestinal mucosal barrier damage. The results of H&E staining of colon tissue revealed that the intestinal cavity of mice in OVX + PBS group was sparser than that in Sham group, and the intestinal space was also significantly enlarged, while the regular and quantitative perfusion of Ph ameliorated the intestinal cavity density of mice in OVX + Ph group. Moreover, the results of IHC and assessment indicated that the expressions of Occludin and ZO-1 (tight junction component proteins) in the gut of mice in OVX + PBS group were reduced, while regular and quantitative perfusion of Ph improved that in OVX + Ph group, indicating that the perfusion of Ph was involved in the repair of damaged intestinal mucosal barrier and optimized the intestinal permeability of mice in OVX + Ph group (Figure 5B–E).

### 3.3. The Perfusion of Ph Decreased the OVX-Mediated Intestinal Inflammation

**Figure 6:**
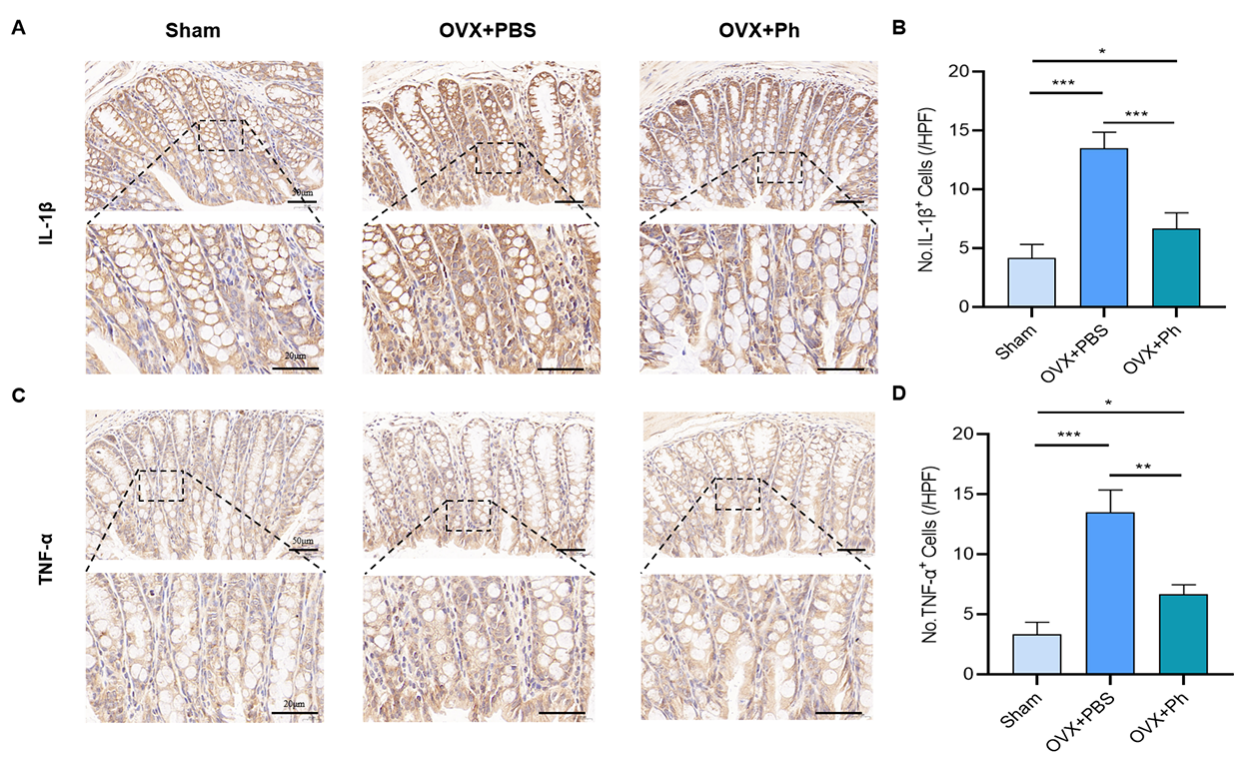
The perfusion of Ph decreased the intestinal inflammation mediated by OVX. (**A**) The IL-1β stained sections of colon tissues. The scale bars represented as 50 μm and 20 μm; (**B**) No. IL-1β^+^ cells; (**C**) TNF-α stained sections of colon tissues. The scale bars represented as 50 μm and 20 μm; (**D**) No. TNF-α^+^ cells. * *p* < 0.05, ** *p* < 0.01, and *** *p* < 0.001 compared with comparable group.

Based on the optimization of intestinal permeability, the results of IHC revealed that the expressions of IL-1β and TNF-α (intestinal inflammatory indicators) of mice in OVX + PBS group were correspondingly higher than that in Sham group, while regular and quantitative perfusion of Ph could decrease the OVX-mediated intestinal inflammation of mice in OVX + Ph group (Figure 6). Hence, combined with above results, it could be acknowledged that the regular and quantitative perfusion of Ph was conducive to the repair of damaged intestinal mucosal barrier in mice, and then laid a foundation for inhibiting the OVX-mediated intestinal inflammation.

### 3.4. The Perfusion of Ph Ameliorated the Disturbance of GM in Mice with OVX-Mediated OP

**Figure 7:**
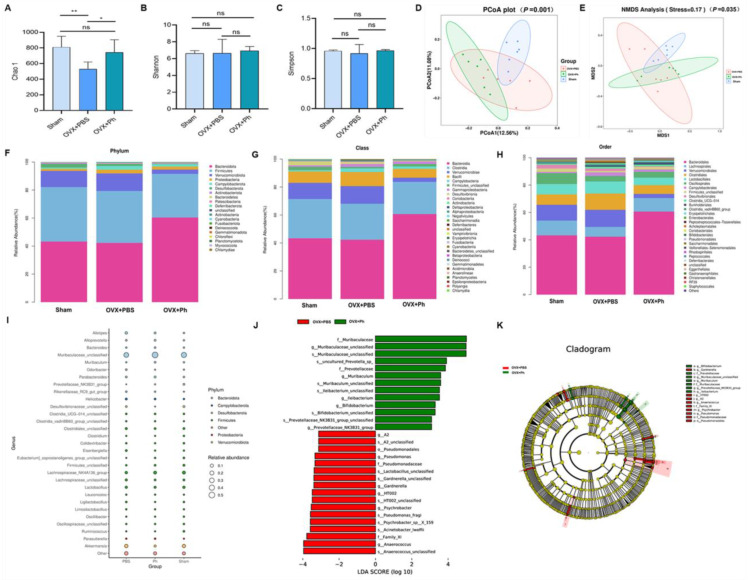
The perfusion of Ph ameliorated the disturbance of GM in mice with OVX-mediated OP. (**A**) Chao 1 index; (**B**) Shannon index; (**C**) Simpson index; (**D**) PCoA plots; (**E**) NMDS analysis plots; (**F**) The composition of GM at phylum level; (**G**) The composition of GM at class level; (**H**) The composition of GM at order level; (**I**) Bubble plots; (**J**) Histogram of LDA value distribution; (**K**) Cladogram. * *p* < 0.05 and ** *p* < 0.01 compared with comparable group; ns no significance.

The fresh feces were collected on the day before mice were sacrificed and after the completion of last perfusion, and immediately stored at −80 °C for the subsequent 16S rRNA high throughput sequencing. As for the α-diversity, the results revealed that there was significant difference regarding Chao1 index between OVX + PBS group and Sham group (*p* < 0.01), and the regular and quantitative perfusion of Ph contributed to restore the richness and diversity of GM of mice in OVX + Ph group (*p* < 0.05, Figure 7A). However, there was no significant difference in terms of the Shannon and Simpson indexes (*p* > 0.05, Figure 7B,C). Based on this, β-diversity analysis was performed to assess the degree of similarity between microbial communities in different groups, and the results of principal coordinates analysis (PCoA) analysis and the non-metric multi-dimensional scaling (NMDS) analysis indicated a distinct clustering of the composition of GM for each group (Figure 7D,E). In details, the microbial community of mice in OVX + PBS group was significantly different with that of mice in Sham group and OVX + Ph group, and the degree of the microbial community aggregation of mice in OVX + Ph group exhibited an equivalent level with that of mice in Sham group. Additionally, the structure and composition of GM at different taxonomic levels were shown in Figure 7F–I and Figure S1A, and the results suggested that the regular and quantitative perfusion of Ph was conducive to improving the disordered structure and composition mediated by OVX. LEfSe analysis (Figure 7J) and cladogram (Figure 7K) were then performed to exhibit significant differences of bacterial taxonomic composition in different groups, and subsequent results suggested that *f_Prevotellaceae*, *s_uncultured_Prevotella_sp_*, *g_Prevotellaceae_NK3B31_group* and *s_Prevotellaceae_NK3B31_group_unclassified* were identified as bacterial taxonomic markers relevant to the perfusion of Ph. In addition, the results of correlation analysis and functional prediction analysis were exhibited in Figure S1B,C.

## 4. Discussion

**Figure 8:**
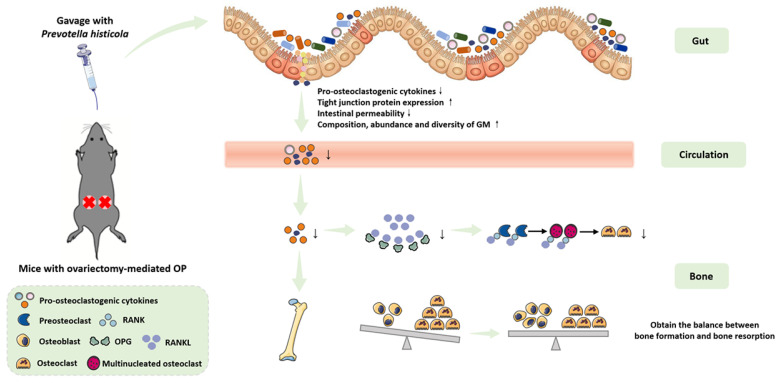
Relevant experimental model diagram and mechanism diagram of this study.

Currently, the aging process of population is unstoppable. With the rapid increase in the number of middle-aged and elderly individuals around the world, OP has become one of the severe public health problems [31]. In recent years, the researches regarding the link between GM and OP has been conducted in full swing [32]. In addition, several human and animal-related studies have reported that the supplementation of probiotics could promote the balance between osteoblast-related bone formation and osteoclast-related bone absorption via a variety of different mechanisms, and thereby inhibiting the bone loss and achieving the goal of prevention and treatment of OP [33,34,35]. In this study, we mainly focused on the protective effects of probiotics (Ph) on bone loss in mice with OVX-mediated OP, and hypothesized that regular perfusion of Ph by gavage can ameliorate the bone loss. Our experimental results showed that regular (once a day for 8 consecutive weeks) and quantitative (200 µL/d) perfusion of Ph (the bacteria that orally gavaged) could improve the bone loss of mice with OVX-mediated OP by repairing intestinal mucosal barrier injury, optimizing intestinal permeability, inhibiting release of pro-inflammatory cytokine, and improving disorder of GM, and blood circulation may act as a mediator of gut-bone crosstalk, which was consistent with the results previously reported by Wang et al. [23]. Furthermore, Figure 8 exhibits the relevant experimental model diagram and mechanism diagram of this study.

On one hand, Ph is widely distributed in the individuals and contributes to decompose the protein and carbohydrate foods. Ph is universally regarded as a kind of bacteria related to healthy plant-based diet, acting as a role of probiotics in human hosts [36]. On the other hand, the decline of Ph is also associated with partial diseases in the human bodies, which can be used as the conditional pathogens to cause diseases, such as periodontitis, multiple sclerosis, rheumatoid arthritis, schizophrenia, OP, and bacterial vaginitis [37,38,39]. The genomic diversity of the strains of Ph might contribute to partially explain the differences in its responses to the daily diets, living habits, and health conditions among human hosts [40]. Meanwhile, more and more in-depth studies are needed to further understand the genetic potential of Ph and its interaction with the hosts and other bacteria, thereby revealing its regulatory properties and potential causal associations to health or diseases.

It is universally acknowledged that the initiating factor of the occurrence and development of postmenopausal OP is regarded as estrogen-deficiency caused by postmenopausal ovarian failure in middle-aged and elderly female [41], while the excessive formation of osteoclasts and subsequent bone resorption effects are the critical pathological alterations formed during this process [42,43]. Moreover, RANK/RANKL/OPG pathway, as one of the significant pathways in the regulation of bone metabolism, participates in the overall process of osteoclasts from differentiation to maturation [44], and the enhanced expression of RANKL and pro-inflammatory cytokines (such as IL-1β and TNF-α) are the main driving factors for the increased generation of osteoclasts [45]. As a result of this, gut is a non-negligible source of the inflammation, and it is also a novel proposition to intervene and relieve the distal targets and organs from the perspective of gut. Based on this, the concept and studies of gut-bone axis have been gradually developed and deepened. In the previous works, our research group also reviewed and summarized the regulatory effects and significance of GM and its metabolites on OP [12], as well as the regulatory effects of exercise [15], and probiotics, prebiotics [17] on GM and OP.

Based on current concepts and studies, it is widely recognized that supplementing proper kind of probiotics with certain amount is essential for reducing the susceptibility to aging-related diseases. Herein, we emphasized the protective effects of probiotics (Ph) on bone loss in mice with OVX-mediated OP, and verified that regular perfusion of Ph by gavage can ameliorate the bone loss. Of note, the modulation of GM through the supplementation of Ph is anticipated to be an encouraging method for the prevention of bone loss. Meanwhile, similar results have been obtained in several previous studies on the application of probiotics. In terms of the human-related studies, Zhao et al. [46] randomly divided 40 individuals with OP into the probiotics group (*n* = 20, received *Bifidobacterium animalis subsp. lactis Probio-M8*) and placebo group (*n* = 20, received placebo material), and then observed that combination of *Probio-M8* and conventional drugs was more effective than the use of conventional drugs alone in the management of OP. Moreover, Morato-Martínez et al. [47] recruited 78 menopausal women with OP in a randomized clinical trial and regularly ingested two different types of nutritional products, and tresults suggested that the consumption of nutritional products containing the probiotics (*Lactobacillus plantarum 3547*) was beneficial to protect bone mass and improve the bone health indicators of menopausal women with OP.

In addition, several animals-related studies have revealed that the supplementation of various types of probiotics could participate in the regulation of bone metabolism through different molecular mechanisms. In this study, we investigated that based on the gut-bone axis and with the involvement of blood circulation, regular perfusion of Ph (by gavage) can improve the bone loss of mice with OVX-mediated OP by repairing intestinal mucosal barrier injury, optimizing intestinal permeability, inhibiting release of pro-inflammatory cytokine, and improving disorder of GM. Moreover, Parvaneh et al. [48] administered the probiotic *Bifidobacterium longum* to the OVX-mediated rats for 16 weeks, and showed that it promoted osteogenesis and inhibited osteoclasis by enhancing the expression of genes (Sparc and Bmp-2), thus reducing the bone loss, and increasing BMD. Lan et al. [49] analyzed protective ability of probiotic *Bifidobacterium lactis BL-99* on the bone of mice with ulcerative colitis induced by sodium glucan sulfate, and the results indicated that *Bifidobacterium lactis BL-99* could prevent the occurrence of OP in mice with ulcerative colitis by remodeling GM and inhibiting production of pro-inflammatory cytokines. Yeom et al. [50] noticed that regularly supplementing OVX-mediated mice with probiotic *Propionibacterium freudenreichii MJ2* isolated from the raw milk could increase the differentiation and mineralization of osteoblasts by enhancing the ratio of OPG/RANKL, thus inhibiting the bone loss caused by estrogen-deficiency. Yang et al. [51] reported that the regular supplementation of two kinds of probiotics (*Lactobacillus plantarum GKM3* and *Lactobacillus paracasei GKS6*) can inhibit the bone loss, and *Lactobacillus paracasei GKS6* exhibited more apparent effects of anti-bone loss than *Lactobacillus plantarum GKM3*. From this, we can recognize that the probiotic supplementation has played a positive role in protecting bone mass and preventing OP, which deserves close attention and deeper exploration by more researchers.

Finally, partial weaknesses and potential advancement of this study need to be identified. Firstly, how to translate the vital results of supplementing Ph to OVX-mediated mice models to prevent the bone loss into clinical application as early as possible is a pending proposition and needs to be explored. Secondly, further researches on the safety, efficacy, dosage, duration, and combination pattern of probiotic products need to be verified by more randomized controlled trials with high evidence level in future. Thirdly, the sample size of OVX-mediated mice model constructed in this study is relatively small, and the subsequent animal experiments with larger sample size are still needed for further verification. Fourthly, in view of the fact that 16S rRNA high-throughput sequencing technique at this current stage is difficult to identify whether the microbiota remain active and lacks the ability to distinguish beyond generic level [52], it is necessary to develop and improve the relevant sequencing approaches to optimize the analysis in the future.

## 5. Conclusions

In general, our experimental results revealed that regular and quantitative perfusion of Ph (the bacteria that orally gavaged) can improve the bone loss in mice with OVX-mediated OP by repairing intestinal mucosal barrier damage, optimizing intestinal permeability, inhibiting release of pro-osteoclastogenic cytokines, and improving disturbance of GM, and blood circulation may act as a mediator of gut-bone crosstalk. Of note, the modulation of GM through the supplementation of Ph is anticipated to be an encouraging method for the prevention of bone loss, although it is still demanded to conduct the animal experimental confirmation and the larger sample size of human prospective clinical trials in the future.

## Data Availability

The data that support the findings of this current study are available on request from the corresponding author.

## Supplementary Materials

The following supporting information can be downloaded at: https://www.mdpi.com/article/10.3390/microorganisms11040950/s1.
