# Supplementary figures and images for: The Preventive Effects of Probiotic Prevotella histicola on the Bone Loss of Mice with Ovariectomy-Mediated Osteoporosis
Source: Microorganisms. 2023 Apr 6;11(4):950. doi: 10.3390/microorganisms11040950 (PMC10146713; doi:10.3390/microorganisms11040950)

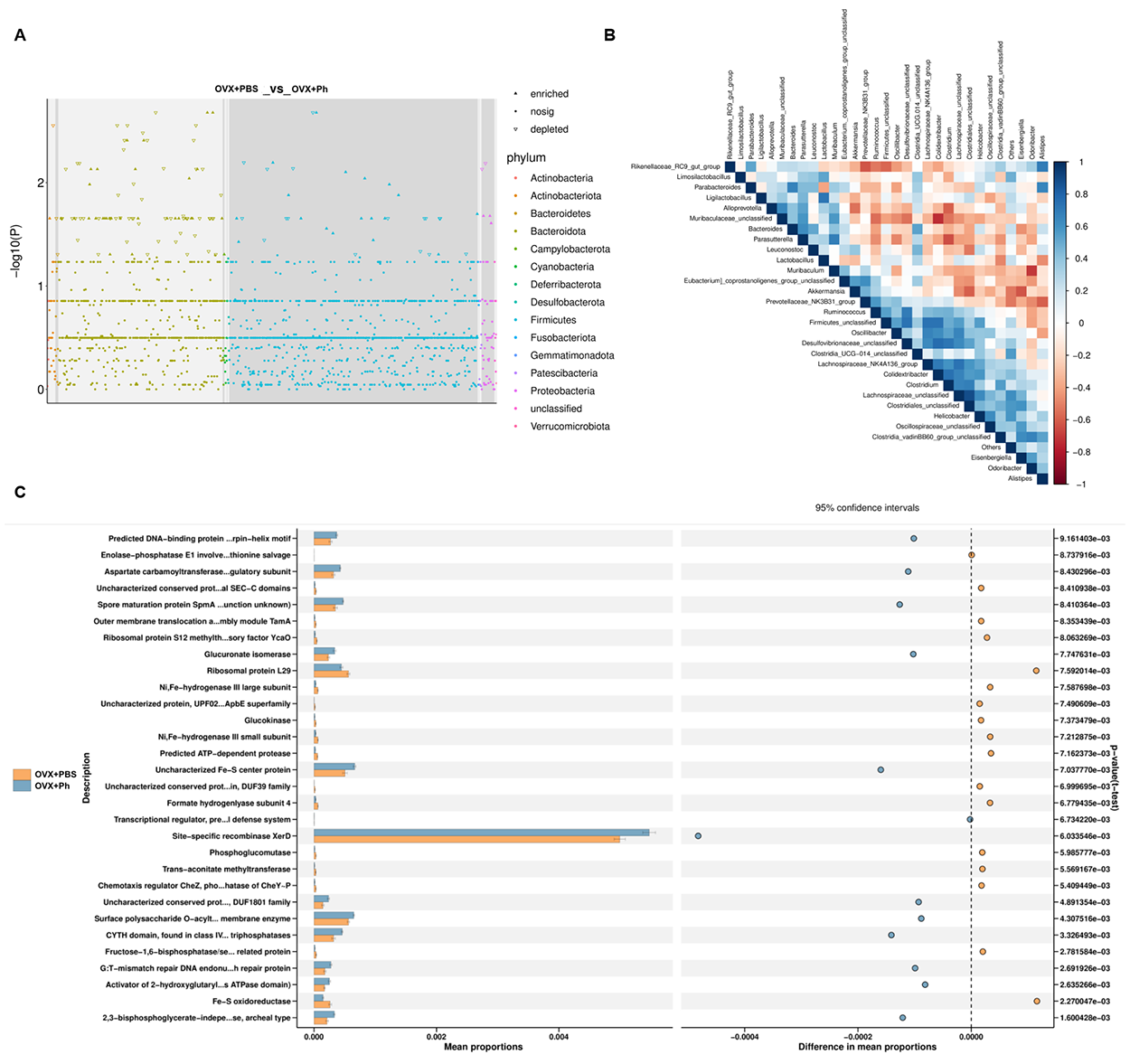

Supplement: Supplementary file 1 [file microorganisms-11-00950-s001.zip › microorganisms-2284708-supplementary.tif]
